# Thermal Stability of Allyl-Functional Phthalonitriles-Containing Benzoxazine/Bismaleimide Copolymers and Their Improved Mechanical Properties

**DOI:** 10.3390/polym10060596

**Published:** 2018-05-29

**Authors:** Mingzhen Xu, Yangxue Lei, Dengxun Ren, Lin Chen, Kui Li, Xiaobo Liu

**Affiliations:** Research Branch of Advanced Functional Materials, School of Materials and Energy, University of Electronic Science and Technology of China, Chengdu 610054, China; leiyangxue@163.com (Y.L.); rendenxun2008@126.com (D.R.); linchen_uestc@163.com (L.C.); leekingue2014@163.com (K.L.)

**Keywords:** allyl-functional phthalonitrile, benzoxazine, bismaleimide, GF-reinforced laminates, crosslinking

## Abstract

Copolymerization is the typical method to obtain the high-performance resin composites, due to its universality and regulation performance. It can be employed among various resin matrices with active groups to obtain the desired structures, and subsequently, the outstanding properties. In this work, the copolymerization between the allyl-functional phthalonitrile-containing benzoxazine resin (DABA-Ph) and 4,4′-bis(Maleimidodiphenyl)methane (BMI) were monitored. The interactions among the active groups including allyl moieties, maleimide, benzoxazine rings and nitrile groups were investigated. Differential scanning calorimetry (DSC) and dynamic rheological analysis (DRA) were used to study the curing behaviors and the processing properties. The possible curing processes were proposed and confirmed by Fourier transform infrared spectroscopy (FTIR). Then, glass fiber-reinforced DABA-Ph/BMI composites were designed, and their thermal-mechanical properties were studied. Results indicated that all the composites exhibited outstanding flexural strength, flexural modulus, and high glass-transition temperatures (*T_g_* > 450 °C). The thermal stability of the composites was studied by thermogravimetry (TGA) and evaluated by the integral program decomposition temperature (IPDT). it is believed that the excellent thermal mechanical properties and outstanding *T_g_* as well as good thermal stability would enable the reinforced copolymer-based laminates to be applied in wider fields.

## 1. Introduction

Fiber-reinforced composite laminates have become more important due to their wide applications in fields from aerospace to energetics [1,2,3]. With the development of science and technology, polymer-based materials have played a vital role in aerospace and marine applications as a result of their ease of processing, high glass-transition temperature, high temperature resistance, excellent mechanical properties, adhesive properties, etc. [4]. Moreover, many properties of polymers can be easily improved or altered by preparing polymer blends or composites [5]. As is well known, the polymer blending, as a kind of technology for generating new materials, has been widely applied in practical fields [6,7]. Various materials usually possess different advantages and they could combine with each other to exhibit their own excellent properties, such as good mechanical properties, processability and high glass-transition temperatures [8,9,10]. Moreover, the properties of blends or copolymers can be easily altered by changing the individual components [8,9,11,12,13]. As a kind of high-performance thermoset resin, bismaleimide (BMI) possesses excellent thermal and mechanical properties, high glass-transition temperature and high modulus [14]. However, it was usually reported that BMI also exhibits bad processability and their polymers exhibit great brittleness, which would limit their applications as structural materials to great extent. Thus, BMI is usually copolymerized with other polymers to improve the final properties [15].

Thermosets derived from phthalonitrile resins have attracted increasing attention due to their excellent glass-transition temperature (*T_g_*), outstanding thermal and thermal-oxidative stability, satisfactory mechanical properties, and superior resistance to moisture, chemicals, and fire [16,17]. Phthalonitrile-based materials possesses the highest heat resistance among the reported matrices while their applications have been limited by the poor processability caused by their high melting points and long curing cycles [18]. Previous researches showed that various curing agents such as metallic salts, organic amines and phenols would trigger the polymerization of phthalonitrile-based resins. Nevertheless, volatilization of curing agents in the process of curing would produce the voids in the networks or composites, which compromised the strengths of the polymer materials. Meanwhile, the decomposition of the remained curing agents in the polymer matrices may reduce the thermo-oxidative stabilities to some extent, which eventually limited the application in industrial areas [19,20,21]. Thus, in our previous work, a novel allyl-functionalized phthalonitrile containing benzoxazine (DABA-Ph) was prepared [22]. The polymerization of DABA-Ph monomer manifested a triple curing pattern corresponding to the sequential polymerization of allyl groups, the ring-forming polymerization of benzoxazine and the ring-opening polymerization of nitrile groups. Compared with that of phthalonitrile-containing benzoxazine (BA-ph), DABA-Ph possesses high crosslinking degrees and excellent thermal stability [22].

Therefore, in this work, BMI was blended with DABA-Ph and their copolymerization behaviors derived from the addition reactions between allyl moieties and maleimide were investigated by DSC and DRA. Moreover, glass fiber-reinforced composite laminates were prepared and their mechanical properties, glass-transition temperatures, crosslinking and thermal stability were also systematically investigated. it is expected that the combination of DABA-Ph and BMI would result in an improved polymer matrix-based composite laminate.

## 2. Experimental

### 2.1. Material

**Scheme 1:**
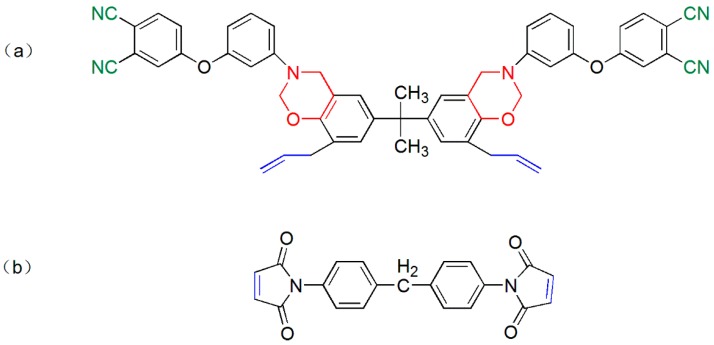
Structures of (**a**) allyl-functional phthalonitriles-containing benzoxazine (DABA-Ph) and (**b**) Bismaleimide (BMI).

DABA-Ph was synthesized in our laboratory and its structure was showed in Scheme 1a [22]. *N*,*N*′-(4,4′-Methylenediphenyl) dimaleimide (BMI) was received from Sichuan University (Chengdu, China), shown in Scheme 1b. Dimethyl sufoxide (DMSO) was received from Tianjin Guangfu Fine Chemical Research Institute (Tianjin, China). *N*,*N*-Dimethylformamide (DMF) and acetone were obtained from Tianjin BODI Chemicals (Tianjin, China). Glass fiber (GF) was E-glass cloth 7682 purchased from Jiangxi Changjia glass fibers Co. Ltd. (Jiangxi, China). All the materials were used as received without further purification.

### 2.2. Preparation of DABA-Ph/BMI Prepolymer

DABA-Ph/BMI prepolymers with various proportions were prepared as follows: moderate DABA-Ph and BMI were mixed in a 100 mL beaker and heated at 160 °C for 30 min with vigorous stirring. The mixture was prepared for the tests of differential scanning calorimetry (DSC). The DABA-Ph/BMI prepolymers were formulated at mole ratios of 1:1, 1:2 and 2:1, and were abbreviated as DABA-Ph/BMI-1/1, DABA-Ph/BMI-1/2, DABA-Ph/BMI-2/1, respectively.

### 2.3. Preparation of DABA-Ph/BMI Copolymers

The DABA-Ph/BMI copolymers were prepared with thermal cured in an air-circulation oven. The detailed heating procedures were as follows: 180 °C–1 h, 200 °C–1 h, 240 °C–2 h, 280 °C–2 h, 320 °C–2 h. The cured DABA-Ph/BMI copolymers were pulverized prior to characterization via FTIR and thermogravimetry analysis.

### 2.4. Fabrication of DABA-Ph/BMI/GF Composite Laminates

The DABA-Ph/BMI/GF composite laminates were prepared via hot-press in a model as follows: DABA-Ph/BMI prepolymers were dissolved in a mixed solvent of DMF and acetone (the volume ratio of DMF and acetone is 1:1) at 80 °C, with vigorous stirring for 2 h. Then, 10 layers of glass fabrics (10 × 10 cm^2^) were impregnated with the viscous solution and dried at 160 °C for 10 min. Next, the dried GF were placed in a stainless steel mold and hot-pressed under a pressure of 20 MPa. The detailed curing procedure was as followed: 180 °C–1 h, 200 °C–1 h, 240 °C–2 h, 280 °C–2 h, 320 °C–2 h. The cured DABA-Ph/BMI/GF composite laminates were tailored for mechanical testing and dynamic mechanical testing.

### 2.5. Characterizations

Differential scanning calorimetric (DSC) analysis was performed by modulated DSC-Q100 (TA Instruments, New Castle, DE, USA) at a heating rate of 10 °C min^−1^ and a nitrogen flow rate of 50 mL min^−1^. The DSC testing of DABA-Ph and DABA-Ph/BMI prepolymer was performed by heating the samples from 50 °C to 350 °C. Dynamic rheological analysis was performed using TA Instruments Rheometer AR-G2 with a frequency of 1 Hz at 180 °C to confirm the curing processability. FT-IR spectra were recorded on a FT-IR8400 S spectrometer (Shimadzu, Kyoto, Japan) in KBr pellets between 4000 and 500 cm^−1^ in air atmosphere. Mechanical properties of the composite laminates were investigated on a SANS CMT 6104 Series. The composite laminates were tailored into strips of about 80 mm in length and 10 mm in width, three strips were needed to gain an average value. DMA in a three-point blending mode was performed on QDMA-800 dynamic mechanical analyzer (TA Instruments) to determine the glass-transition temperatures (*T_g_*). The storage modulus and Tan δ were investigated at a frequency of 1 Hz and amplitude of 20 mm, and the samples (dimensions 30 × 10 × 2 mm^3^) were heated from 50 °C to 400 °C at a temperature ramp of 5 °C min^−1^. The morphology of the fractured surfaces of the composites was observed by scanning electron microscope (SEM, JSM25900LV) operating at 20 kV. Thermogravimetry (TGA) was performed on a TGA Q50 with a heating rate of 20 °C/min (under nitrogen or air) and a purge of 40 mL/min from 50 °C to 600 °C, TA Instruments, (New Castle, DE, USA).

## 3. Results and Discussion

### 3.1. Designing of DABA-Ph/BMI Pre-Polymers and Their Curing Behaviors

**Scheme 2:**
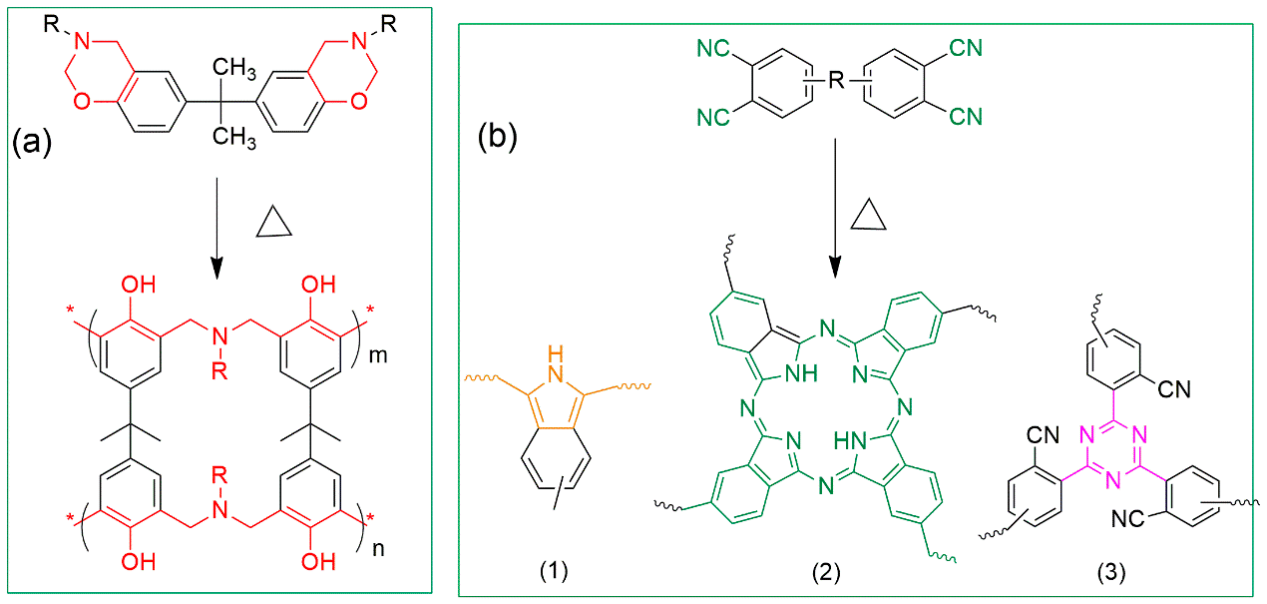
(**a**) The ring-opening polymerization of benzoxazine and (**b**) the ring-forming polymerization of nitrile groups: (1) the resulted isoindole; (2) the phthalocyanine ring and (3) the triazine ring.

In previous work, the allyl-functionalized phthalonitrile containing benzoxazine (DABA-Ph, shown in Scheme 1a) exhibits lower melting viscosity. Their final polymer possesses higher glass-transition temperature and thermal stability, due to the higher crosslinking degree derived from the extra polymerization of allyls groups [19,22]. Moreover, the ring-opening polymerization of benzoxazine rings (shown in Scheme 2a) and ring-forming polymerization of nitrile groups (shown in Scheme 2b) also contributed to the outstanding thermal stability [19,20]. Thus, in this work, the DABA-Ph was selected as the based matrices to modify the traditional thermosetting resins, which is expected to provide kinds of novel high-performance thermosets. Considering of the allyls groups present in the structure of DABA-Ph, BMI (shown in Scheme 1b) was selected to copolymerize with allyl groups. It was widely reported that the copolymerization between BMI and allyls contributed to the brittleness of BMI polymers [23,24]. In this work, the copolymers of BMI and DABA-Ph were designed and investigated through monitoring the curing behaviors.

**Figure 1:**
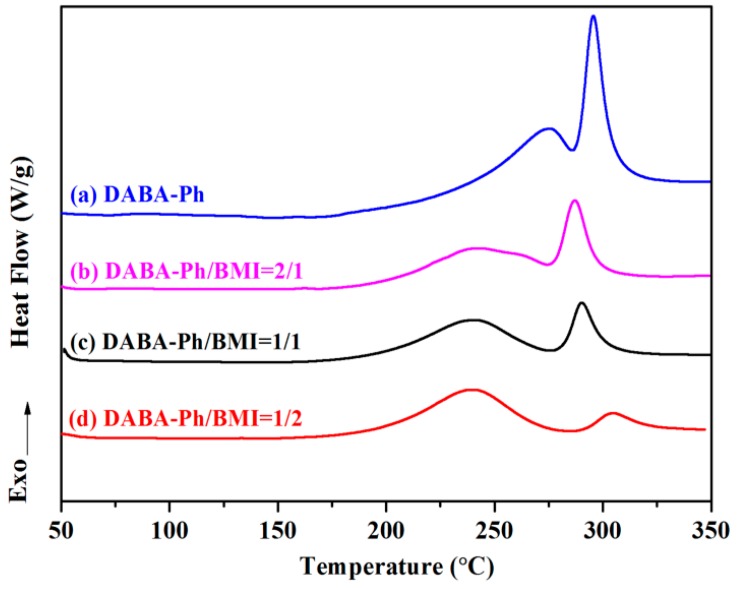
DSC curves of DABA-Ph/BMI copolymers with various proportions.

The curing behaviors of DABA-Ph/BMI prepolymers were investigated with the testing of DSC. Figure 1 showed the DCS curves of DABA-Ph/BMI with various proportions. It is obvious that all of the prepolymers exhibited at least dual-stages exothermic transition in the range from 50 to 350 °C without endothermic peaks, corresponding to the curing processes [22]. For the curve of DABA-Ph, triple-stage curing processes were reported and the wide exothermic band from 170 to 270 °C was assigned to the overlapped addition polymerization of allyl groups and the ring-opening polymerization of benzoxaine rings. Moreover, the polymerization of the allyl moieties was weak and prior to that of the ring-opening polymerization of oxazine rings [22]. With the introduction of BMI (Figure 1b), the addition polymerization of allyl moieties occurred in the range from 170 to 250 °C, and the exothermic peak intensity increased obviously, indicating the significant copolymerization between allyl moieties and BMI. The exothermic peak of the ring-opening of oxazine rings at about 265 °C was also be observed, indicating that it was partly overlapped by the wide bands of the copolymerization process but was barely be affected. Also, the ring-forming polymerization of nitrile groups was also not significantly affected, which can be confirmed by the exothermic enthalpy shown in Table 1.

**Figure 2:**
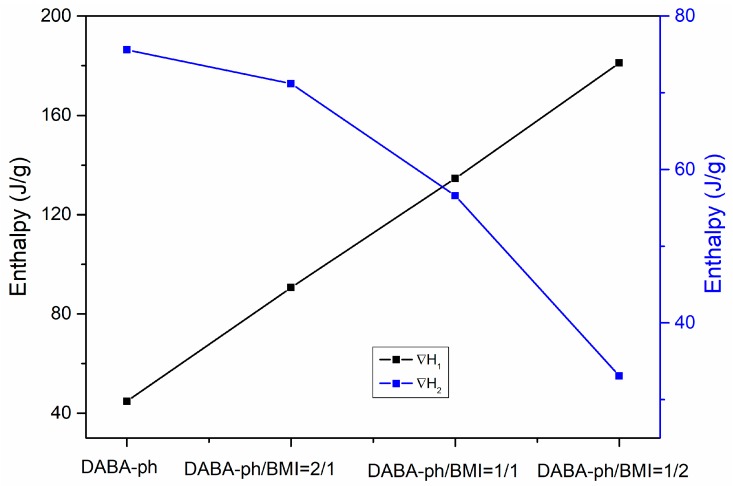
Enthalpy of DABA-Ph/BMI copolymers with various proportions.

As mentioned above, the obvious double-stage exothermic peak was assigned to the overlap of the addition reaction of allyl moieties and ring-opening reaction of oxazine, and the ring-forming polymerization of nitrile groups. Figure 2 showed the exothermic enthalpy of the double-stage exothermic peaks as function of the content of BMI for the various DABA-Ph/BMI blends. It was obvious that with increasing the proportions of BMI, the exothermic peak in the range from 170 to 275 °C increased and the corresponding exothermic enthalpy increased, indicating the increasing of the polymerization rates. However, the polymerization of nitrile groups decreased with increasing the content of BMI, which was verified by both the exothermic peak position and the exothermic enthalpy, indicating the inhibition of BMI on the ring-forming polymerization of nitrile groups. Considering of the ring-forming polymerization of nitrile groups in this system, the phenolic hydroxyl and amine structures derived from the ring-opening of benzoxazine rings were the key initiators and accelerators [20]. In comparison with that of pristine DABA-Ph, the copolymerization between allyl moieties and BMI would increase the steric hindrance during the polymerization process of nitrile groups. The active hydrogen would be partly involved into the addition reaction of BMI [25,26]. Thus, the polymerization rate and degree of nitrile groups would weaken accordingly with increasing the content of BMI.

### 3.2. Processing Properties of DABA-Ph/BMI Pre-Polymers

**Figure 3:**
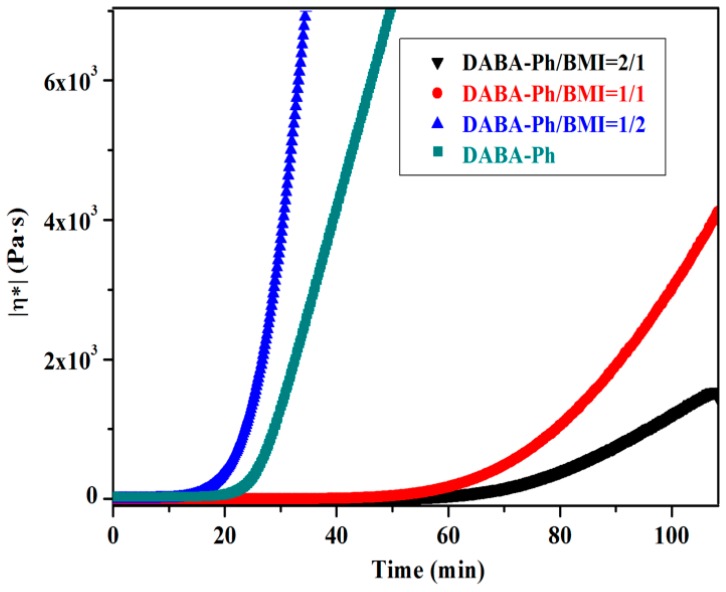
Gelation time at 180 °C for DABA-Ph/BMI pre-polymers with various content of BMI.

Dynamic rheological analysis on the various DABA-Ph/BMI prepolymers was performed to characterize the processability of the blends and reveal their curing behaviors. Figure 3 showed the rheological behaviors of the DABA-Ph monomer and various DABA-Ph/BMI prepolymers. The complex viscosities (η*) of DABA-Ph/BMI systems are measured as a function of time at 180 °C and the detailed data are collected in Table 2. It can be seen that the gel-time of DABA-Ph monomer at 180 °C is about 22.7 min, while the gel time of DABA-Ph/BMI-2/1, DABA-Ph/BMI-1/1 and DABA-Ph/BMI-1/2 are 61.4 min, 52.1 min and 18.3 min, respectively. The gel time is the key parameter during the processing of the resin matrix. The DABA-Ph/BMI-1/2 system exhibits a relatively short gel time, indicating that the introduction of BMI could improve the curing reactivity of DABA-Ph/BMI systems. Meanwhile, the DABA-Ph/BMI-1/1 and DABA-Ph/BMI-1/2 systems in 180 °C exhibit a relatively slow increase in viscosity and the tendency of increase is relatively gentle. It can be ascribed to the fact that the promotion effects on the copolymerization between DABA-Ph and BMI would be determined by the amount of BMI. Moreover, the gelling viscosities of the pre-polymers with various BMI contents collected from the DRA tests are presented in Table 2. The high gelling viscosities of DABA-Ph and DABA-Ph/BMI-1-2 are observed, due to their high rate of polymerization. The gelling viscosities of the DABA-Ph/BMI-1-1 and DABA-Ph/BMI-2-1 pre-polymers are relatively lower than that of DABA-Ph and DABA-Ph/BMI-1-2. It can be attributed to their low pre-polymerization. The results are in close agreement with the gel time of the pre-polymers. Thus, it can be concluded that 180 °C is a proper processing temperature for DABA-Ph monomer and the polymerization degree of DABA-Ph/BMI system could be controlled by tuning the content of BMI at 180 °C. The results provide an appropriate processing temperature and time for DABA-Ph/BMI pre-polymers and it would be suitable for the industrial applications.

### 3.3. The Structure and Possible Polymerization Processes of DABA-Ph/BMI Pre-Polymers

**Figure 4:**
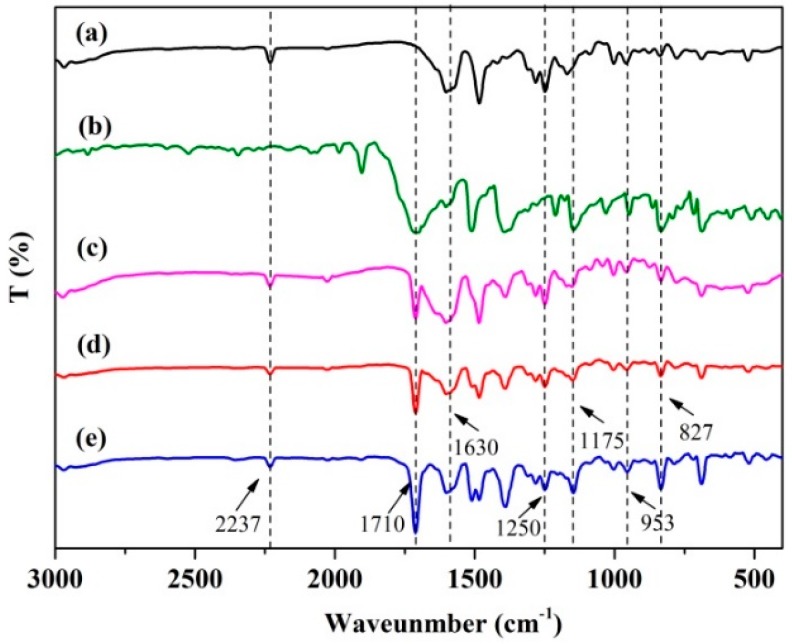
FTIR spectra of (**a**) DABA-Ph monomer; (**b**) BMI monomer; (**c**) DABA-Ph/BMI-2/1 prepolymer; (**d**) DABA-Ph/BMI-1/1 prepolymer and (**e**) DABA-Ph/BMI-1/2 prepolymer treated at 200 °C.

Figure 4 showed the FT-IR spectra of the DABA-Ph/BMI blends treated at 200 °C with various BMI contents, in which the pre-polymers with various contents of DABA-Ph and BMI treated at 200 °C are presented. The characteristic absorption peaks was listed as follows: FTIR (potassium bromide, KBr, cm^−1^): 2237 cm^−1^ (-CN), 1710 cm^−1^ (imide, C=O), 1630 cm^−1^ (allyl, C=C), 1250 cm^−1^ (phenolic hydroxyl, -OH), 1175 cm^−1^ (succinimide, C-N-C), 953 cm^−1^ (oxaxine ring), 827 cm^−1^ (maleimide, C=C) [27,28]. Generally speaking, the characteristic absorption peaks of the active groups would change due to the polymerization between DABA-Ph and BMI. The characteristic absorption peak of oxazine ring (953 cm^−1^) decreases and the characteristic absorption peak of active phenolic hydroxyl (1250 cm^−1^) becomes apparent (Figure 4a). It can be ascribed to the ring-opening polymerization of oxazine rings at 200 °C, which would generate active hydroxyl groups. Moreover, in comparison with the absorption peaks in Figure 4c–e, it is observed that with increasing the content of BMI, the characteristic absorption peak of phenolic hydroxyl decreases accordingly, due to the electrophilic addition reactions between BMI and phenolic hydroxyl. It can be confirmed by the fact that the increase of absorption peak at 1175 cm^−1^ (succinimide, C-N-C) and the decrease of absorption peak at 827 cm^−1^ (maleimide, C=C) (shown in Figure 4b–e). Additionally, the absorption peak of allyl group (1630 cm^−1^) was not visible, which agrees with the results of DSC that the allyl groups react at about 160 °C. The characteristic absorption peak at 2237 cm^−1^ is assigned to the nitrile groups and the peak at 1710 cm^−1^ is assigned to the C=O imide. Thus, it can be concluded that the ene reactions between DABA-Ph and BMI occur at 200 °C.

**Scheme 3:**
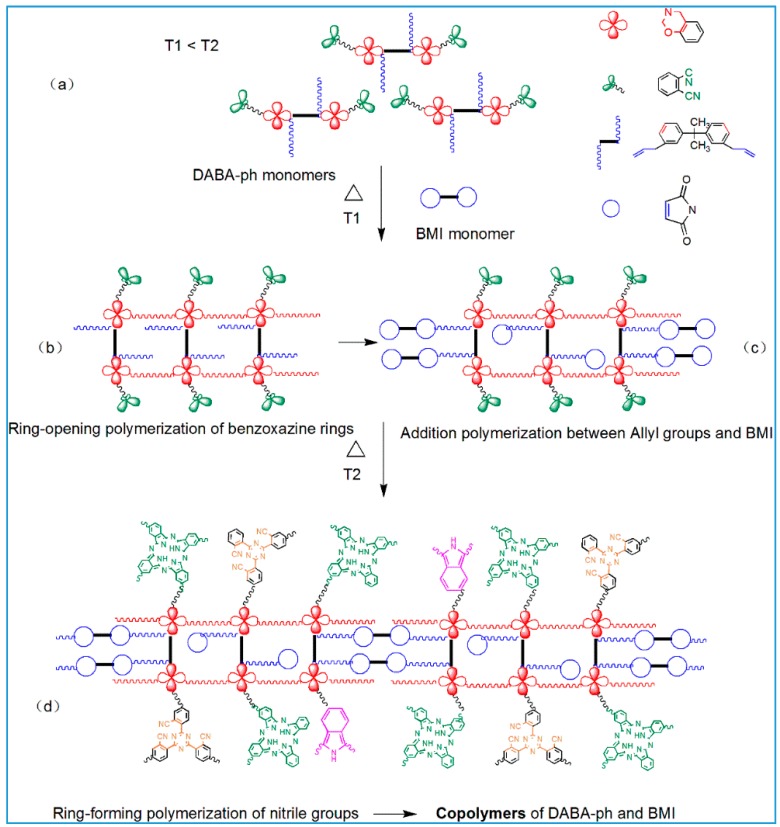
Possible polymerization reaction of DABA-Ph/BMI system: (**a**) structure of DABA-Ph and BMI monomer; (**b**) ring-opening polymerization of oxazine rings; (**c**) addition reaction between allyls and BMI; (**d**) the possible structure of the final composites.

Thus, the possible copolymerization processes of DABA-Ph and BMI can be summarized as shown in Scheme 3. The ring-opening polymerization of oxazine rings and the addition polymerization of allyl groups occurs almost at the same temperature range (shown in Scheme 3a,b). During the ring-opening polymerization of benoxazine rings, the generated active hydrogens and amine structures would sequentially accelerate the copolymerization between oxazine rings and BMI, resulting from the ene reactions. Finally, with persistently arising the treated temperature, the ring-forming polymerization of nitrile groups occurred in the presence of active hydrogens and amine structures. Finally, crosslinking network composites can be obtained due to the coexistence of phthalocyanine, triazine rings and isoindole structures.

### 3.4. Mechanical Properties of the Composite Laminates and Their Interface Property

**Figure 5:**
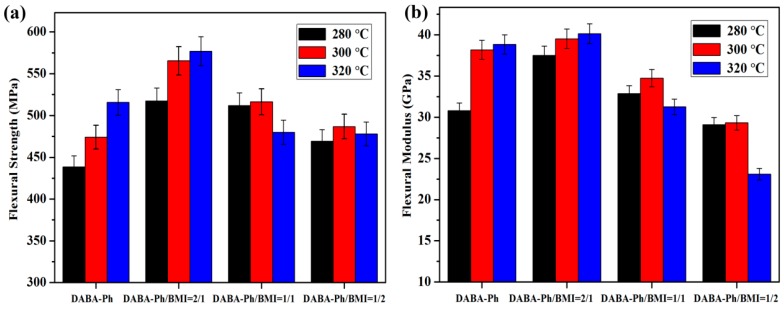
Mechanical properties of DABA-Ph/BMI/GF composite laminates at different temperatures: (**a**) flexural strength; (**b**) flexural modulus.

The mechanical properties of DABA-Ph/BMI/GF composite laminates prepared at various temperatures were evaluated and presented in Figure 5. Compared with that of DABA-Ph/BMI composite laminate, it is observed that the DABA-Ph/BMI-2/1 composite laminate possess the maximum flexural strength and flexural modulus. However, with the increase of BMI content, the flexural strength and flexural modulus decrease. It was reported that the enhancement of flexural strength and modulus can be attributed to the network structures of the matrix and the interfacial adhesion between the matrix and fibers. In DABA-Ph/BMI system, the ene reactions between DABA-Ph and BMI, the homo-polymerization of BMI and the polymerization of allyl moieties would all affect the strength of the matrix. The addition of a small amount BMI could improve the flexural strength and modulus effectively. However, brittleness is a major limitation of pristine BMI, leading to inferior mechanical properties. Thus, persistent increasing the content of BMI, the flexural strength and modulus would decrease due to the brittleness of BMI itself. Furthermore, treatment temperatures also show significant effects on the flexural strength and modulus. Increasing the treatment temperature, the flexural strength and modulus increase correspondingly [29]. It also confirms the fact that increasing the crosslinking degree would improve the mechanical properties to some extent.

**Figure 6:**
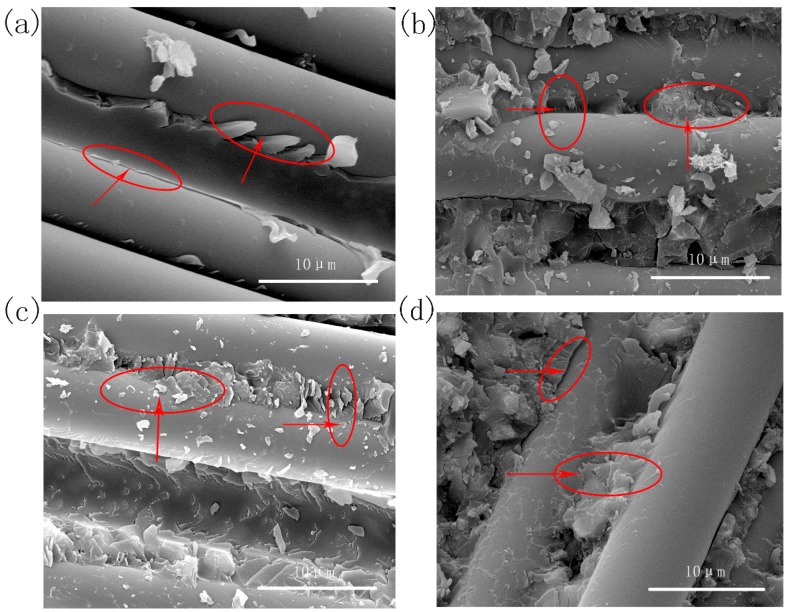
The morphology of the fracture surface for DABA-Ph/BMI/GF composite laminates with various content of BMI treated at 320 °C: (**a**) DABA-Ph; (**b**) DABA-Ph/BMI-1/2; (**c**) DABA-Ph/BMI-1/1 and (**d**) DABA-Ph/BMI-1/2.

The fracture surface of the composites with various content of BMI treated at 320 °C was studied and shown in Figure 6. it is obvious that all of the fibers are embedded into the matrix and no pulled out fibers were in sight. Figure 6a shows the fracture surface of DABA-Ph/GF composites. The smooth fracture surface of the matrix is obvious and the interfacial layers (which are labeled by the red circles and arrows) between the matrix and fibers are also observed. It indicates the relative brittleness of the DABA-Ph matrix and the weak interfacial adhesion between DABA-Ph and fibers. Figure 6b presents the fracture surface of DABA-Ph/BMI/GF composites. The strong interfacial adhesion is observed from the rough fracture surface and the matrix coated on GFs. Moreover, the matrix existing between the fibers (labeled with the red circles and arrows) adheres strongly to the fibers. No obvious cracks are observed, indicating the outstanding adhesion between the DABA-Ph/BMI matrix and the fibers. In comparison with that of DABA-Ph matrix, the difference of the fracture surface can be attributed to the difference of the wetting ability of the matrix. In the system of DABA-Ph/BMI, the copolymerization between DABA-Ph and BMI would generate various kinds of polar groups including the active hydroxyl, amine structures and other polar intermediates, which helped to improve the interface adhesion. With increasing the content of BMI, the fracture surfaces of the composites are presented in Figure 6c,d. The similar surfaces of the composites indicate the similar interfacial adhesion. No cracks and stripping can be observed for both of the composites shown in (c) and (d), revealing the improved interface compatibility of the DABA-Ph/BMI matrix and the fibers. In sum, the fracture surfaces of the various composites indicate that the interfacial adhesion can be improve by the introduction of BMI, in well agreement with the improved mechanical properties of the various composites with BMI.

### 3.5. Dynamic Thermal Mechanical Properties of the DABA-Ph/BMI/GF Composite Laminates

**Figure 7:**
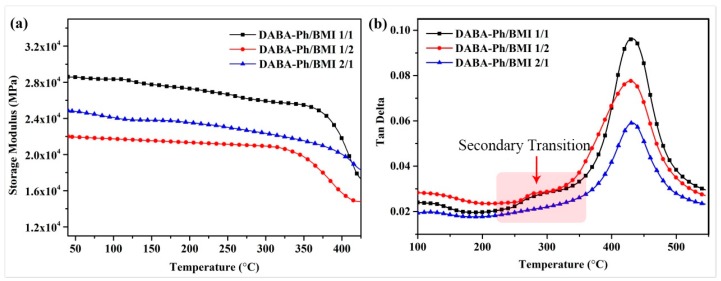
Dynamic mechanical properties of DABA-Ph/BMI composite laminates: (**a**) storage modulus (**b**) Tan δ.

The dynamic thermal mechanical properties of DABA-Ph/BMI composite laminates were investigated. The storage modulus and tan delta of various DABA-Ph/BMI/GF composites laminates were presented in Figure 7a,b, respectively. Meanwhile, the initial storage modulus and glass-transition temperature (*T_g_*) are listed in Table 3. From Figure 7a, it can be seen that all the initial storage moduli of DABA-Ph/BMI/GF composites are high (>20,000 MPa). Among them, the DABA-Ph/BMI-1/1 possesses the highest storage modulus. According to the researches, storage modulus usually represents the ability of a material to store elastic energy, which can be determined by the stiffness of the matrix itself and the interface adhesion between the matrix and fibers [30]. Thus, the high modulus of the composites can be attributed to the completed polymerization of the matrix and the improved interfacial adhesion between the matrix and the fibers. Then, obvious decline of the modulus for all of the composites appears at about 350 °C to 400 °C. This phenomenon can be assigned to the glass transition of the matrix.

To further investigate the glass-transition process of the matrix, Figure 7b shows the Tan delta (Tan δ) of various DABA-Ph/BMI/GF composites laminates. As is well known, *T_g_* is the peak temperature of tan δ and it depends on the crosslinking degree and the motion of molecular segments. From Figure 7b, it is observed that the *T_g_* of all the DABA-Ph/BMI/GF composites are above 430 °C, which are higher than that of other traditional resins, including polybenzoxazine, phenolic, epoxy resins and so on. The excellent *T_g_* could be attributed to the complete polymerization of DABA-Ph/BMI matrix at 320 °C, which would result in a high crosslinking degree. Moreover, the crosslinking degree could be improved by the copolymerization of DABA-Ph and BMI, including the ring-opening polymerization of benzoxazine, the addition polymerization of allyl moieties, the polymerization of the nitrile groups and the homo-polymerization of BMI. In addition, the secondary transitions are also observed at 250 °C to 350 °C, which can be assigned to the relaxation of the crosslinking network [31]. Increasing the content of BMI, the secondary transition weakens, indicating the polymerization of BMI possesses stronger intermolecular forces, which would hinder the relaxation and movement of the molecular chains.

### 3.6. Thermal Stability of the DABA-Ph/BMI/GF Composite Laminates

**Figure 8:**
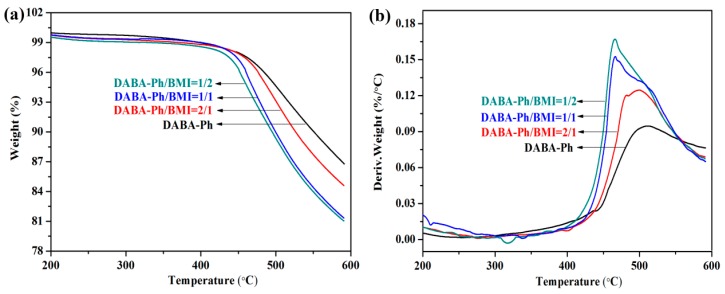
(**a**) TGA curves and (**b**) DTG curves of DABA-Ph/BMI copolymers with various proportions treated at 320 °C.

Thermal stabilities of DABA-Ph/BMI cured polymers were evaluated by TGA under N_2_ atmosphere at a heating rate of 20 °C/min, shown in Figure 8. The main results were summarized in Table 4, including the initial decomposition temperatures (*T_i_*), the decomposition temperatures at weight loss of 5% (*T_5%_*), the maximum decomposition temperatures (*T_m_*), and the char yield at 600 °C. As shown in in Figure 8a and Table 4, DABA-Ph composites possess excellent thermal stability and its *T_5%_* is 497.3 °C. It is attributed to the relatively high crosslinking degree due to the sequential polymerization of allyl moieties, oxazine rings, and nitrile groups. With the introduction and increasing the content of BMI, the *T_5%_* of DABA-Ph/BMI systems decreases. It can also be explained by the copolymerization between DABA-Ph and BMI. During the copolymerization, various intermediates composed with alkane segments appear in the network, which would damage the thermal stability of the composites to some extent.

To understand the thermal degradation characteristics of the composites with various content of BMI, the differential presentation of integral TGA curves (DTA) is shown in Figure 8b. All the plots of the composites with BMI show double peaks at about 480 °C–520 °C, corresponding to the double decomposition processes of the DABA-Ph/BMI composites. For the DABA-Ph composite, the DTG curve shows a single peak at about 500 °C, indicating to the decomposition of the principle component. In comparison with that of DABA-Ph, the double decomposition processes of DABA-Ph/BMI composites are assigned to the decomposition of the copolymerization intermediates and the main crosslinking network composed with the heteroaromatic structures. Above of all, the DABA-Ph/BMI/GF composite laminates presented satisfactory thermal and thermo-stabilities [32].

**Figure 9:**
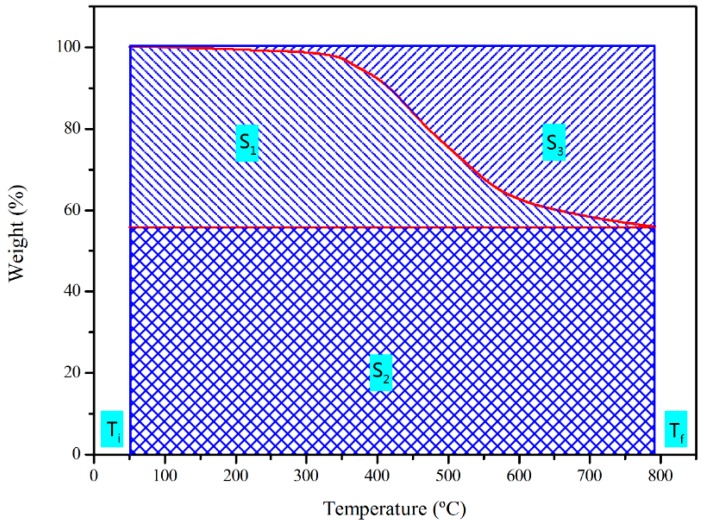
Schematic representation of S1, S2 and S3 for A* and K*.

To further verify the thermal stability, the integral program decomposition temperature (IPDT) was used as an evaluation, which is a common method to evaluate the inherent thermal stability of different materials. It is related to the volatile compounds of polymer materials, and the results are not affected by the experimental conditions and particle size, shape, and appearance of the samples. IPDT consists of Formula (1) [11,33].

(1) $$IPDT=A^{*}K^{*}\times \left(T_{f}-T_{i}\right)+T_{i}$$

where *A* is the area ratio of the total experimental curve defined by the total TGA thermogram curve. *T_i_* is the initial experimental temperature and *T_f_* is the final experimental temperature. In this study, *T_i_* and *T_f_* were 50 °C and 790 °C, respectively. *A* and *K* can be calculated by Equations (2) and (3). The values of S1, S2, and S3 are determined by Figure 9.

(2) $$A^{*}=\frac{S_{1}+S_{2}}{S_{1}+S_{2}+S_{3}}$$

(3) $$K^{*}=\frac{S_{1}+S_{2}}{S_{1}}$$

The IPDT values of DABA-Ph/BMI-1/2, DABA-Ph/BMI = 1/1, DABA-Ph/BMI-2/1 and DABA-Ph are respectively 3479 °C, 3877 °C, 2953 °C, 2545 °C. This is because the complete polymerization between DABA-Ph and BMI, including the ring-opening polymerization of oxazine rings, the addition polymerization of allyl moieties and BMI, the ring-forming polymerization of nitrile groups. The polymerization would provide rich aromatic nucleus and heterocyclic ring, thereby enhancing the inherent thermal stability of the composites.

## 4. Conclusions

In this work, DABA-Ph/BMI blends and their curing behaviors were studied in detail. Results indicated the copolymerization between DABA-Ph and BMI can be initiated easily at about 200 °C and completed at about 320 °C. The copolymerization of DABA-Ph/BMI system presented two stages of polymerizations, assigning to the additional polymerization of allyl moieties and maleimide moieties the ring-forming polymerization of nitrile groups, respectively. The fiber-reinforced DABA-Ph/BMI composites were prepared and treated at various temperatures. The investigation of the mechanical properties of the composites indicated that the DABA-Ph/BMI-2/1 composite exhibits the highest flexural strength and modulus, due to the perfect copolymerization between DABA-Ph and BMI. The flexural strength and modulus of the composites increased with increasing the heat-treated temperatures, indicating the complete polymerization of the DABA-Ph/BMI matrix would contribute to improving the mechanical properties. Combined with the results of DMA and SEM, it can be concluded that the increased mechanical can be attributed to the improvement of the crosslinking degree and the enhancement of the interface adhesion between fibers and the matrix. Moreover, the composites possess outstanding *T_g_* (>450 °C) and excellent thermal stability, which would enable them to apply in fields such as aerospace and warships.

## Figures and Tables

**Table 1:** Copolymerization behaviors of DABA-Ph/BMI prepolymers.

Sample	*T_i_* (°C)	*T_p_* (°C)	*T_p_*′ (°C)	*T_p_*″ (°C)	ΔH_1_ (J/g)	ΔH_2_ (J/g)
DABA-Ph	227.75	272.8	-	295.6	44.8	75.6
DABA-Ph/BMI-2-1	197.41	238.2	263.3	287.1	90.6	71.2
DABA-Ph/BMI-1-1	199.35	239.4	-	290.3	134.6	56.6
DABA-Ph/BMI-1-2	198.55	239.7	-	304.7	181.1	33.1

*T_i_*: the initial curing temperatures. *T_p_*: the peak curing temperatures.

**Table 2:** Viscosity and Gel-time of DBPA-Ph/BMI Prepolymer at 180 °C.

Sample	Initial Viscosity (Pa·s)	Gel-Time (min)	Gelling Viscosity (Pa·s)
DABA-Ph	2.471	22.7	417.2
DABA-Ph/BMI-2-1	0.447	61.4	63.26
DABA-Ph/BMI-1-1	0.175	52.1	62.11
DABA-Ph/BMI-1-2	0.113	18.3	146.1

**Table 3:** Storage modulus and glass-transition temperature (*T_g_*) of DABA-Ph/BMI composite laminates.

Sample	Initial Storage Modulus at 50 °C (MPa)	*T_g_* (°C)
DABA-Ph ^#^	28,975	382
DABA-Ph/BMI-2-1	24,781	430.1
DABA-Ph/BMI-1-1	28,556	430.9
DABA-Ph/BMI-1-2	21,944	430.5

Note: ^#^ Which were reported in our previous work [22].

**Table 4:** Thermal stability of DABA-Ph/BMI copolymers treated at 320 °C.

Sample	*T_i_* (°C)	*T_5%_* (°C)	*T_m_* (°C)	Char Yield at 600 °C (%)
DABA-Ph	450.7	497.3	502.2	86.4
DABA-Ph/BMI-2-1	452.8	484.2	496.5	84.5
DABA-Ph/BMI-1-1	443.3	466.4	462.7	81.3
DABA-Ph/BMI-1-2	439.5	459.6	454.1	80.7

*T_i_*: the initial decomposition temperatures. *T_5%_*: the decomposition temperatures. *T_m_*: the initial decomposition temperatures.

## References

[1] Bulgakov B.A., Sulimov A.V., Babkin A.V., Afanasiev D.V., Solopchenko A.V., Afanaseva E.S., Kepman A.V., Avdeev V.V. (2017). Flame-retardant carbon fiber reinforced phthalonitrile composite for high-temperature applications obtained by resin transfer molding. Mendeleev Commun..

[2] Bulgakov B.A., Sulimov A.V., Babkin A.V., Kepman A.V., Malakho A.P., Avdeev V.V. (2017). Dual-curing thermosetting monomer containing both propargyl ether and phthalonitrile groups. J. Appl. Polym. Sci..

[3] Derradji M., Ramdani N., Zhang T., Wang J., Gong L.-D., Xu X.-D., Lin Z.-W., Henniche A., Rahoma H.K.S., Liu W.-B. (2017). Thermal and mechanical properties enhancements obtained by reinforcing a bisphenol-a based phthalonitrile resin with silane surface-modified alumina nanoparticles. Polym. Compos..

[4] Xu M., Liu M., Dong S., Liu X. (2013). Design of low temperature self-cured phthalonitrile-based polymers for advanced glass fiber composite laminates. J. Mater. Sci..

[5] Ding Y., Lu B., Wang P., Wang G., Ji J. (2018). PLA-PBAT-PLA tri-block copolymers: Effective compatibilizers for promotion of the mechanical and rheological properties of PLA/PBAT blends. Polym. Degrad. Stab..

[6] Augustine D., Mathew D., Nair C.P.R. (2015). Phthalonitrile resin bearing cyanate ester groups: Synthesis and characterization. RSC Adv..

[7] Augustine D., Mathew D., Nair C.P.R. (2015). Mechanistic and kinetic aspects of the curing of phthalonitrile monomers in the presence of propargyl groups. Polymer.

[8] Guo H., Zou Y., Chen Z., Zhang J., Zhan Y., Yang J., Liu X. (2012). Effects of self-promoted curing behaviors on properties of phthalonitrile/epoxy copolymer. High Perform. Polym..

[9] Xu Q., Zeng M., Chen J., Zeng S., Huang Y., Feng Z., Xu Q., Yan C., Gu Y. (2018). Synthesis, polymerization kinetics, and high-frequency dielectric properties of novel main-chain benzoxazine copolymers. React. Funct. Polym..

[10] Liu C., Sun M., Zhang B., Zhang X., Li J., Wang L., Xue G., Zhao M., Song C., Li Q. (2017). Preparation and properties of acetylene-terminated benzoxazine/epoxy copolymers. React. Funct. Polym..

[11] Zabihi O., Khodabandeh A., Mostafavi S.M. (2012). Preparation, optimization and thermal characterization of a novel conductive thermoset nanocomposite containing polythiophene nanoparticles using dynamic thermal analysis. Polym. Degrad. Stab..

[12] Deveci S., Antony N., Eryigit B. (2018). Effect of carbon black distribution on the properties of polyethylene pipes—Part 1: Degradation of post yield mechanical properties and fracture surface analyses. Polym. Degrad. Stab..

[13] Huang W., He W., Long L., Yan W., He M., Qin S., Yu J. (2018). Highly efficient flame-retardant glass-fiber-reinforced polyamide 6T system based on a novel DOPO-based derivative: Flame retardancy, thermal decomposition, and pyrolysis behavior. Polym. Degrad. Stab..

[14] Xiong X., Zhou L., Ren R., Ma X., Chen P. (2018). Thermal, mechanical properties and shape memory performance of a novel phthalide-containing epoxy resins. Polymer.

[15] Harsha A.P., Tewari U.S., Venkatraman B. (2003). Solid particle erosion behaviour of various polyaryletherketone composites. Wear.

[16] Sastri S.B., Keller T.M. (1998). Phthalonitrile cure reaction with aromatic diamines. J. Polym. Sci. Part A Polym. Chem..

[17] Keller T.M., Price T.R. (2006). Amine-Cured Bisphenol-Linked Phthalonitrile Resins. J. Macromol. Sci. Part A Chem..

[18] Cao G.P., Chen W.J., Liu X.B. (2008). Synthesis and thermal properties of the thermosetting resin based on cyano functionalized benzoxazine. Polym. Degrad. Stab..

[19] Xu M., Luo Y., Lei Y., Liu X. (2016). Phthalonitrile-based resin for advanced composite materials: Curing behavior studies. Polym. Test..

[20] Xu M., Ren D., Chen L., Li K., Liu X. (2018). Understanding of the polymerization mechanism of the phthalonitrile-based resins containing benzoxazine and their thermal stability. Polymer.

[21] Xu M.Z., Jia K., Liu X.B. (2015). Effect of bisphenol-A on the structures and properties of phthalonitrile-based resin containing benzoxazine. Express Polym. Lett..

[22] Xu M., Jia K., Liu X. (2016). Self-cured phthalonitrile resin via multistage polymerization mediated by allyl and benzoxazine functional groups. High Perform. Polym..

[23] Wang K., Wang Y., Chen P., Xia L., Xiong X. (2018). Novel Bismaleimide Resins Modified by Allyl Compound Containing Liquid Crystalline Structure. Adv. Polym. Technol..

[24] Ge M., Miao J.-T., Yuan L., Guan Q., Liang G., Gu A. (2018). Building and origin of bio-based bismaleimide resins with good processability, high thermal, and mechanical properties. J. Appl. Polym. Sci..

[25] Radue M.S., Varshney V., Baur J.W., Roy A.K., Odegard G.M. (2018). Molecular Modeling of Cross-Linked Polymers with Complex Cure Pathways: A Case Study of Bismaleimide Resins. Macromolecules.

[26] Li W., Li Q., Wang X., Lu S., Wang B. (2018). The role of maleic anhydride functionalized graphene oxide in improving the interfacial properties of carbon fibre/bismaleimide composites. Polym. Int..

[27] Zhao Y., Xu Y., Xu Q., Fu F., Zhang Y., Endo T., Liu X. (2018). Significant Improvement on Polybenzoxazine Toughness Achieved by Amine/Benzoxazine Copolymerization-Induced Phase Separation. Macromol. Chem. Phys..

[28] Xu M., Yang X., Zhao R., Liu X. (2013). Copolymerizing Behavior and Processability of Benzoxazine/Epoxy Systems and Their Applications for Glass Fiber Composite Laminates. J. Appl. Polym. Sci..

[29] Chua J., Tu Q. (2018). A Molecular Dynamics Study of Crosslinked Phthalonitrile Polymers: The Effect of Crosslink Density on Thermomechanical and Dielectric Properties. Polymers.

[30] Augustine D., Mathew D., Nair C.P.R. (2015). End-functionalized thermoplastic-toughened phthalonitrile composites: Influence on cure reaction and mechanical and thermal properties. Polym. Int..

[31] Laskoski M., Schear M.B., Neal A., Dominguez D.D., Ricks-Laskoski H.L., Hervey J., Keller T.M. (2015). Improved synthesis and properties of aryl ether-based oligomeric phthalonitrile resins and polymers. Polymer.

[32] Hidalgo J., Jimenez-Morales A., Torralba J.M. (2013). Thermal stability and degradation kinetics of feedstocks for powder injection moulding—A new way to determine optimal solid loading?. Polym. Degrad. Stab..

[33] Qian Y., Wei P., Jiang P., Zhao X., Yu H. (2011). Synthesis of a novel hybrid synergistic flame retardant and its application in PP/IFR. Polym. Degrad. Stab..

